# Cutaneous, mucocutaneous and visceral leishmaniasis in Sweden from 1996–2016: a retrospective study of clinical characteristics, treatments and outcomes

**DOI:** 10.1186/s12879-018-3539-1

**Published:** 2018-12-07

**Authors:** Hedvig Glans, Leif Dotevall, Sara Karlsson Söbirk, Anna Färnert, Maria Bradley

**Affiliations:** 1 Department of Infectious Diseases, Karolinska University Hospital, Stockholm, Sweden; 2 Division of Dermatology and Venerology, Department of Medicine Solna, Karolinska Institutet, Stockholm, Sweden; 3 Department of Communicable Disease Control Region, Västra Götaland, Gothenburg, Sweden; 4 Division of Infection Medicine, Department of Clinical Sciences, Faculty of Medicine, Lund University, Lund, Sweden; 5 Division of Infectious Diseases, Department of Medicine Solna, Karolinska Institutet, 17176 Stockholm, Sweden; 6 Department of Dermatology, Karolinska University Hospital, Stockholm, Sweden

## Abstract

**Background:**

Leishmaniasis is a neglected and poorly reported parasitic infection transmitted by sand flies in tropical and subtropical regions. Knowledge about leishmaniasis has become important in non-endemic countries due to increased migration and travel. Few studies of the clinical management of cutaneous, mucocutaneous and visceral leishmaniasis in non-endemic regions have been published to date. In this study, we aimed to evaluate patient characteristics, clinical manifestations and treatments of leishmaniasis in Sweden, over a 20-year period.

**Methods:**

A retrospective observational nationwide study was performed using medical records of patients diagnosed with leishmaniasis in Sweden from 1996 to 2016. Cases with culture and polymerase chain reaction verified leishmaniasis were identified at the Public Health Agency of Sweden.

**Results:**

In total, 165 cases of leishmaniasis were diagnosed from 1996 to 2016. Medical records from 156 patients (95%) were available for review and included in the study. Cutaneous leishmaniasis was the dominant manifestation (*n* = 149, 96%), and in 66 patients (44%) cutaneous leishmaniasis was due to *Leishmania tropica*. Other manifestations were mucocutaneous (*n* = 4, 3%), visceral (*n* = 2, 1%) and post-kala-azar dermal leishmaniasis (*n* = 1, 1%). During this time period, the number of cases increased, especially after 2013. Most patients (*n* = 81, 52%) were migrants who were infected in their countries of origin (from 2013 to 2016, mainly Syria or Afghanistan). Other groups were Swedish tourists (25%) and returning workers (13%). The time from collection of the diagnostic sample to the start of treatment was less than one month in 81 (66%) patients and under three months in 124 patients (96%). Among the 149 patients with cutaneous leishmaniasis, 125 patients received antileishmanial treatment, and in 88 of these patients (70%) cure was achieved, regardless of treatment.

**Conclusions:**

The number of leishmaniasis cases diagnosed in Sweden increased between 1996 and 2016, mainly in migrants from endemic countries. Although leishmaniasis is a rare disease in Sweden, patients appear to be diagnosed early and treated according to current European guidelines, resulting in an overall high cure rate.

**Electronic supplementary material:**

The online version of this article (10.1186/s12879-018-3539-1) contains supplementary material, which is available to authorized users.

## Background

Leishmaniasis, a vector-borne disease caused by the intracellular parasite *Leishmania*, is defined by the World Health Organization (WHO) as a neglected tropical disease [1]. *Leishmania* infection has a broad range of clinical manifestations: cutaneous (CL), mucocutaneous (MCL), visceral (VL), post-kala-azar dermal (PKDL), and diffuse cutaneous leishmaniasis, depending on the *Leishmania* species and the immunological response of the patient [2]. Leishmaniasis is now endemic in at least 102 tropical and subtropical countries and regions [3, 4].

For decades, pentavalent antimonial was the gold standard treatment regardless of the *Leishmania* species. Due to treatment failure and relapse, possibly caused by resistance among certain subtypes, as well as drug toxicity, new treatments have been introduced. Currently, several alternatives are available and recommended depending on the clinical manifestation, subtype/species, and geographical region [5].

Experience in managing leishmaniasis is often limited in non-endemic regions, which may lead to a delay in diagnosis and unfavourable treatment outcomes [6]. In Sweden, patients are rare and seldom encountered in clinical practice. There is no domestic leishmaniasis in Sweden because the vector is absent; however, the number of imported leishmaniasis cases has increased in recent years [7]. Management of leishmaniasis in Sweden has not previously been described, and there are no Swedish guidelines for treatment. Hence, treatment guidelines published by other European countries and the USA have been used [8, 9].

In this study, we assess patient characteristics, clinical presentation and treatments of leishmaniasis in Sweden from 1996 to 2016, with aim to evaluate the treatment outcomes and optimise the management of leishmaniasis in a non-endemic setting.

## Methods

### Study population

All cases of microbiologically confirmed leishmaniasis in Sweden between January 1996 and December 2016 were included in the study. Cases were identified at the National Reference Laboratory of the Public Health Agency of Sweden, which is the only laboratory in Sweden that conducts subtyping of *Leishmania*. Medical records were retrieved from treating hospitals around Sweden.

### Laboratory procedures

*Leishmania* parasites were detected through culture and/or polymerase chain reaction (PCR) of tissue or bone marrow samples. Patients with only positive serological testing or microscopy findings were assessed as unconfirmed cases and were not included due to limitations in interpreting the results.

Species typing was performed using different methods during the study period. From 1996 to 2010, monoclonal antibodies from the WHO were used to define the parasite species. From 2010 to 2016, restriction fragment length polymorphism (RFLP)-PCR of the parasite IST1 gene was used [10, 11]. In 2016, heat shock protein 70 (hsp70) PCR was introduced for species typing [12]. Since 2010, the ability in differentiating between species within the same complex has increased, especially regarding the *L. donovani* and *L. braziliensis* complexes*.*

### Data collection

Personal identification number on confirmed leishmaniasis cases were collected from databases at Public Health Agency of Sweden. Medical records were retrieved from the hospital clinics where the patients were treated, by linking their personal identification number. Data were collected using a standardized protocol regarding demographic, epidemiological, clinical, and laboratory parameters, management, treatment, and outcome. Information regarding side effects and “definitions” were obtained through information from medical records.

### Ethical approval

Ethical approval was obtained from the Central Ethical Review Board in Stockholm (2015/2162–31).

### Definitions

*Cutaneous leishmaniasis (CL)* was defined as skin lesions, usually papules that evolve into nodules and ulcers and heal with scarring [1, 13].

*Mucocutaneous leishmaniasis (MCL)* was defined as ulceration of the mucous membranes of the nose, mouth, or pharynx, which may cause destruction of the soft tissue in the nasal and oral regions [1].

*Visceral leishmaniasis (VL)* was defined as generalized involvement of the reticuloendothelial system, such as the spleen, bone marrow, liver, or lymph nodes [1, 13].

*Cure* was defined as the absence of clinical relapse for 6 months after treatment [1].

*Relapse* was defined as the recurrence of a lesion after the lesion had healed, without any known new exposure to the parasite.

*Treatment failure* was defined as the absence of clinical signs of reepithelialisation in the lesion during or within 2 months after treatment.

*Time to first contact with healthcare system* was defined as the time from the first symptoms to the first visit at a hospital clinic in Sweden.

*Time of diagnosis* was defined as the date when the diagnostic sample was collected, as this was thought to be the date closest to the first clinical suspicion of leishmaniasis.

### Statistical analysis

Statistical analyses were performed using Stata software version 13. Logistic regression was used to estimate the probability of relapse (binary outcome: 0 = no, 1 = relapse and treatment failure). The regression model included the treatment as an independent variable. The treatments (sodium stibogluconate (SS), liposomal amphotericin (LA), cryotherapy, and fluconazole) were included in the regression model. SS treatment outcome was used as the reference.

When the subtype *L. tropica* or migrant status was added to the above model, the remaining variables did not change by more than 10% (data not shown); therefore, *L. tropica* and migrant status were excluded from the final regression model.

## Results

**Fig. 1:**
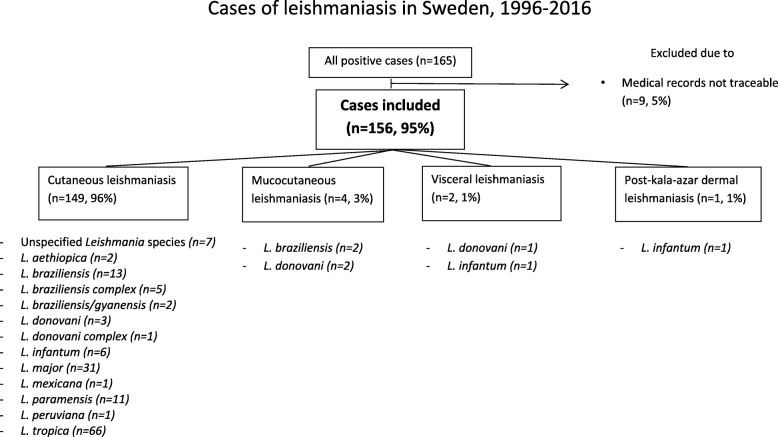
Distribution of clinical manifestations and *Leishmania* subtypes in patients with leishmaniasis in Sweden, 1996–2016

In total, 165 cases of leishmaniasis were diagnosed at the National Reference Laboratory from 1996 to 2016, and medical records from 156 patients (95%) were available for review and included in the study. CL was the dominant manifestation (*n* = 144, 96%). Other manifestations were MCL, VL and PKDL. The most common species were *L. tropica, L. major and L. braziliensis/L. braziliensis* complex. In seven cases, subtyping analysis was not possible due to the small amount of DNA extracted from the samples or limitations in the analysis over time (Fig. 1).

**Fig. 2:**
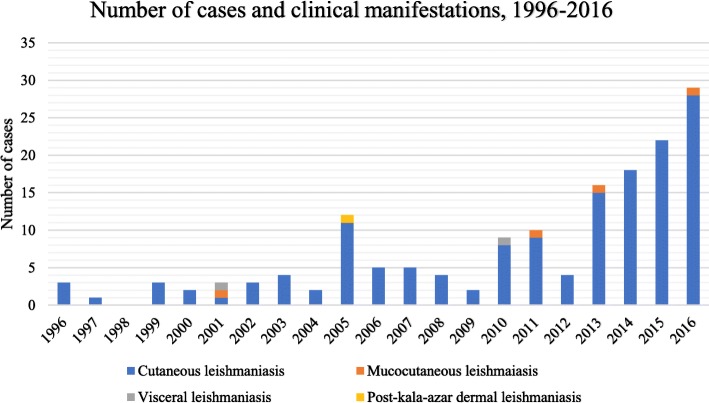
Number of cases of leishmaniasis and types of clinical manifestations, 1996–2016

The annual number of leishmaniasis cases increased during the period 1996–2016, especially from 2013 to 2016 (Fig. 2). The patients were mainly adolescents or young men (Table 1). Migrants were the largest group of patients, followed by tourists and returning workers, including volunteers and military personnel (Table 1). The military personnel came from the Swedish Armed Forces and were infected in Afghanistan in 2010, as previously described [14]. Immunosuppression among the patients was rare (Table 1).

**Table 1:** Main characteristics of patients with leishmaniasis diagnosed in Sweden, 1996–2016 (*n* = 156)

	Cases *n* = 156	Percent %		
Gender				
Male	96	*62*		
Age (years)				
< 4	12	*7*		
5–14	25	*16*		
15–24	29	*19*		
25–34	30	*19*		
35–44	19	*12*		
45–54	12	*8*		
55–64	20	*13*		
> 65	9	*6*		
Reason for stay in endemic region				
Migrants	81	*52*		
Months in Sweden:				
0–3	22	*27*		
4–6	21	*26*		
7–9	8	*10*		
10–12	7	*9*		
> 12	10	*12*		
No data	13	*16*		
Tourists	39	*25*		
Working/living abroad	8	*5*		
Swedish Armed Forces	12	*8*		
Visiting relatives and friends	14	*9*		
Temporarily visiting Sweden	2^a^	*1*		
HIV				
Yes	1	*1*		
No	47	*30*		
No data	108	*69*		
Immunosuppressive treatment				
Yes	4	*3*		
No	152	*97*		
Time to first health contact at hospital clinic (*n* = 150)				
Months	Migrants	*(%)*	Non-migrants	*(%)*
1	1	*1*	2	*3*
2	2	*3*	15	*21*
3	10	*13*	20	*28*
4	5	*6*	9	*12*
5–6	16	*21*	12	*17*
7–8	13	*17*	6	*8*
9–10	5	*6*	3	*4*
> 10	26	*33*	5	*7*
Time from diagnostic sample collection to start of treatment (*n* = 133)				
Months				
1	81	*61*		
2	28	*21*		
3	15	*11*		
> 4	6	*5*		
No date	3	*2*		

^a^one from Ethiopia and one from Iran

The most common countries of origin of infection were Syria (*n* = 50, 32%) and Afghanistan (*n* = 30, 19%), followed by Iran (*n* = 11, 7%) (Additional file 1: Figure S1).

The time from start of symptoms to first visit at hospital varied between migrants and the other groups. Among non-migrants, 37 patients (51%) sought medical care for symptoms and lesions within the first three months, whereas migrants often reported having had symptoms before arriving in Sweden (Table 1).

Treatment was started within the first three months after the diagnostic sample was collected in 124 (93%) of the patients, and 81 patients (61%) were treated within the first month (Table 1).

### Cutaneous leishmaniasis

**Fig. 3:**
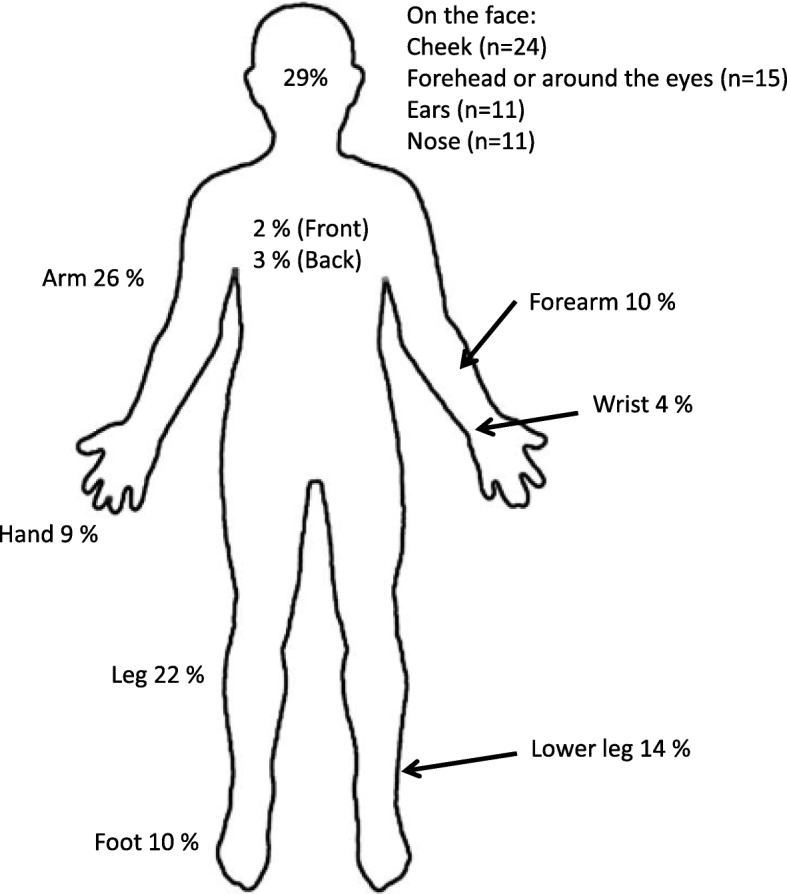
Distribution of lesions (one or more) in patients with cutaneous leishmaniasis (CL). Most patients with CL had a single lesion (*n* = 83, 56%), followed by two lesions (*n* = 34, 23%) and three to five lesions (*n* = 24, 16%). Only 5% of patients had more than five lesions

CL lesions were mainly located on exposed surfaces of the skin, e.g., the face (Fig. 3).

### Cutaneous leishmaniasis treatment and outcomes

Among the 149 patients with CL, 125 (84%) patients received treatment, and 21 (14%) patients healed without treatment. Data on treatment and outcome were missing for 3 patients. Systemic or local SS, LA, cryotherapy, and fluconazole were most frequently used (Table 2).

**Table 2:** Treatment of cutaneous leishmaniasis. *Leishmania* species in cutaneous leishmaniasis cases, with first-line and second-line treatments and outcomes; data from Sweden, 1996–2016. Sodium stibogluconate (SS), liposomal amphotericin (LA), intralesional (il), intravenous (iv)

*Leishmania* species	Treatment		No of cases	Cured	Treatment failure	Relapse	Allergic reaction	Lost to follow-up after treatment
	First-line	Second-line						
*L.tropica*	SS (il)		3	2	0	1	0	0
		SS + paromomycin ointment	1	–	–	1	–	–
	SS (iv)		13	9	1	3	0	0
		LA	3	–	1	2^a^	1	–
		Paromomycin ointment	1	–	–	–	1	–
	SS (iv) + Cryotherapy		2	2	0	0	0	0
	LA		15	6	5	2	1 ^b^	1^c^
		SS	4	–	3	1	–	–
		LA	1	–	1	–	–	–
		Cryotherapy	1	–	1	–	–	–
		Paromomycin ointment	1	–	–	1	–	–
	LA + Cryotherapy		4	2	1	0	1	0
		SS	1	–	–	–	1	–
		Cryotherapy	1	–	1	–	–	–
	Cryotherapy		17	16	0	1	0	0
		SS	1	–	–	1	–	–
	Fluconazole		4	0	4	0	0	0
		SS	2	–	2	–	–	–
		LA	1	–	1	–	–	–
		Cryotherapy	1	–	1	–	–	–
	Paromomycin ointment		1	0	1	0	0	0
		SS (iv)	1	–	1	–	–	–
*L.major*	SS (il)		7	7	0	0	0	0
	SS (iv)		4	4	0	0	0	0
	SS + Cryotherapy		1	1	0	0	0	0
	LA		1	1	0	0	0	0
	Fluconazole		8	5	3	0	0	0
		SS (iv)	1	–	1	–	–	–
		Cryotherapy	1	–	1	–	–	–
		Miltefosine	1	–	1	–	–	–
	Cryotherapy		3	3	0	0	0	0
*L. braziliensis/braziliensis* complex	SS (il)		2	0	2	0	0	0
		LA	1	–	1	–	–	–
		Ketaconazole	1	–	1	–	–	–
	SS (iv)		10	7	2	0	1	0
		LA	2	–	1	–	1	–
		Meglumine antimoniate	1	–	1	–	–	–
	LA		4	2	2	0	0	0
		SS (iv)	2	–	2	–	–	–
*L. panamensis*	SS (iv)		7	7	0	0	0	0
	Ketoconazole		1	1	0	0	0	0
	Miltefosine		1	1	0	0	0	0
	Paromomycin ointment		1	1	0	0	0	0
Unspecified *Leishmania* species	SS (il)		2	2	0	0	0	0
	SS (iv)		1	0	1	0	0	0
		LA	1	–	1	–	–	–
	Fluconazole		1	1	0	0	0	0
*L. infantum*	LA		4	3	1	0	0	0
		LA	1	–	1	–	–	–
	Photodynamic therapy		1	1	0	0	0	0
*L. donovani*	Fluconazole		2	1	1	0	0	0
		LA	1	–	1	–	–	–
	Cryotherapy		1	1	0	0	0	0
*L. aethiopica*	SS (iv)		1	0	1	0	0	0
		Cryotherapy	1	–	1	–	–	–
*L. braziliensis/gyanensis*	LA		1	1	0	0	0	0
*L. mexicana*	SS (iv)		1	0	0	0	1	0
		Ketoconazole	1	–	–	–	1	–
*L. peruviana*	SS (iv)		1	1	0	0	0	0

a continued to relapse, both after LA as a single treatment and after LA in combination with cryotherapy; after five years, the lesions are still not cured

b was lost to follow-up after a reaction during the first infusion with LA

c died during follow-up, of medical reasons unrelated to *Leishmania* infection or anti-*Leishmania* treatment

SS was used in 55 patients (44%); 16 patients (29%) received SS intralesionally, and 39 patients (71%) received SS intravenously. The next most common treatments were LA (*n* = 29, 23%), cryotherapy (*n* = 21, 17%), and fluconazole (*n* = 15, 12%) (Table 2). Five patients (3%) were treated with other regimens. For 17 patients (11%), the lesions healed without treatment (Table 2). For five patients (3%), no documentation of outcome could be found; four of the five patients left Sweden before treatment had been started, and the fifth patient died during follow-up, after treatment with LA, of reasons unrelated to *Leishmania* infection or treatment.

Intralesional SS cured 13 patients (81%), and intravenous SS cured 31 patients (79%). Thirteen patients with treatment failure, relapse or side effects after SS were cured after second-line treatment. Two patients relapsed after second-line treatment (Table 2).

LA as a first-line treatment cured 15 patients (52%). Thirteen patients did not respond to treatment or relapsed but were cured after second-line treatment (Table 2).

Cryotherapy was given in 21 patients, and 20 of these patients (95%) were cured (Table 2). The number of sessions varied from one to eight and the duration of the cryotherapy sessions was rarely documented in the medical records.

Comparison of the four most common treatments, SS, LA, cryotherapy, and fluconazole, using logistic regression showed fewer treatment relapses and failures in the cryotherapy group than in the SS group, although this difference was not statistically significant (OR 0.18, 95% CI 0.02–1.47, *p* value 0.110). More treatment failures and relapses were also found in the LA (OR 2.46, 95% CI 0.91–6.69, *p* value 0.077) and fluconazole groups (OR 4.10, 95% CI 1.23–13.59, *p* value 0.021) than in the SS group, but these differences were not all statistically significant.

### Side effects of cutaneous leishmaniasis treatment

Patients receiving local treatment (*n* = 16) with SS and/or cryotherapy (*n* = 25) reported pain during intralesional administration and/or cryotherapy and up to two hours after intralesional administration. There were no records of secondary bacterial infections or other adverse events after local treatments.

Systemic treatment with SS, as a first-line or second-line treatment, was given to 49 patients. This treatment resulted in elevated liver enzymes (1.1–5.7 μkat/L, median 2.15 μkat/L) in 31 patients (63%) and elevated pancreatic amylase (1.05–12 μkat/L, median 3.6 μkat/L) in 29 patients (59%) during both shorter (10 days) and longer (20 days) treatment regimens, but all laboratory levels normalized after treatment. No arrhythmia was reported.

Among the 46 patients given LA as a first- or second-line treatment, eight patients (17%) reported a reaction during administration of the first dose, with symptoms of chest pain, palpitations, dizziness, respiratory disorders and malaise. Three patients were able to complete the treatment when given a low infusion rate, two patients tolerated the treatment in combination with hydrocortisone, three patients were treated with SS instead, and one patient was lost to follow-up after the first infusion with LA. Eight patients (17%) reacted with elevated creatinine levels (109–277 μmol/L, median 162 μmol/L). The treatment was paused, the patients were given extra intravenous fluid, and when the creatinine level had normalized, treatment was continued.

General side effects, such as myalgia, headache, nausea, tiredness, and/or abdominal pain, were reported in 15 patients (33%) given LA and in 25 patients (51%) given intravenous SS.

### Mucocutaneous leishmaniasis

Four patients were diagnosed with MCL. All patients had symptoms and clinical manifestations of active mucosal involvement and were examined by otolaryngologists. Two patients were infected with *L. braziliensis,* and two patients were infected with *L. donovani*. The patients were infected in Africa (*n* = 1), Latin America (*n* = 2), or Europe (*n* = 1).

Two patients with *L. braziliensis* were administered SS as a first-line treatment. One patient was cured, and the other patient was cured after second-line treatment with LA. The two patients infected with *L. donovani* were treated with LA, and both relapsed. One of these patients had nose bleeds, but the main problem was mucosal thickening of the soft palate, which caused no damage but complicated swallowing and eating. Infection with actinomycosis was also found, and treatment with doxycycline was started. Despite this treatment, the clinical symptoms recurred after four months; extended LA treatment was given for an additional seven days, and the patient was then cured.

The other patient with MCL due to *L. donovani* had lung disease treated with methotrexate and prednisolone and had problems breathing and swallowing. Due to immunosuppressive treatment, an extended LA regimen with a total dose of 60 mg/kg was given. No relapse was recorded during the first year after treatment.

### Visceral leishmaniasis

Two patients were diagnosed with VL and infected in Spain (*n* = 1) and Greece (*n* = 1). One patient with *L. donovani* had no comorbidity and was cured after treatment with LA for 14 days. The other patient, who was infected with *L. infantum,* was co-infected with HIV and had several relapses after treatment. After first-line treatment with LA, miltefosine was given for 28 days. After a couple of months, a new relapse occurred, and a new attempt with LA treatment once every week was initiated. Initially, combination treatment with interferon gamma-1b (Imukin®) was used, but that treatment was stopped due to interferon gamma-1b-induced pancreatitis. The patient continued to deteriorate, and immunoglobulin (KIOVIG®) was administered without any improvement in VL. The patient later died due to kidney and bone marrow failure interpreted to be due to the HIV infection. The CD4 count was low regardless of HAART, and the immunological status was never restored throughout this period.

### Post-kala-azar dermal leishmaniasis

PKDL was diagnosed in a patient previously treated with LA for VL in Montenegro, before coming to Sweden. Symmetrical, non-ulcerating, nodular lesions appeared on both sides of the face one year after treatment. PCR from a skin biopsy revealed *L. infantum*. The patient was treated with an additional course of LA for three weeks and was cured. There was no relapse during the four years of follow-up.

### Patients on immunosuppressive therapies

Three patients with rheumatic diseases were treated with methotrexate, etanercept or infliximab therapies, and one patient, described as having MCL, with a chronic lung disease, was treated with methotrexate and prednisolone. All patients tested negative for HIV. The three patients with rheumatic diseases had CL; two were infected with *L. infantum* and one was infected with *L. tropica.* All patients were treated with LA, and their immunosuppressive therapies were withdrawn during treatment. The two patients with *L. infantum* were cured, but the patient with *L. tropica* showed some improvement during treatment but no re-epithelization. Ten days after leishmaniasis treatment ended, treatment with tumour necrosis factor (TNF) inhibitors was restarted. Within a couple of weeks, the lesion started to progress again. Second-line treatment with cryotherapy was used, and the lesion healed.

## Discussion

This retrospective study describes clinical characteristics, treatment and outcomes of leishmaniasis in Sweden from 1996 to 2016. CL was the dominant manifestation. Other manifestations were mucocutaneous (*n* = 4, 3%), visceral (*n* = 2, 1%) and post-kala-azar dermal leishmaniasis (*n* = 1, 1%). Two cases of MCL due to *L. donovani* were diagnosed among our patients. Mucocutaneous manifestations are rare for *L. donovani* and only described a few times in the litterature [15, 16].

The number of leishmaniasis cases increased during the study period, especially between 2013 and 2016, when the number of migrants from highly endemic areas, such as Syria and Afghanistan, increased in Sweden [17]. In previous studies from European countries, the distribution of cases was different; with migrants being a minority, and travellers and those visiting friends and relatives being the majority [18, 19]. We found that many of the migrants had been in Sweden for up to six months before they had their first medical examination. This “patient delay” was not observed in the other groups, which generally sought healthcare within the first three months after the onset of symptoms. Some of the migrants had already received treatment for leishmaniasis, but many had not sought any healthcare for their manifestations before they reached Sweden. This observation highlights the importance of identifying and treating migrants during their migration through Europe and before they reach Sweden. Nonetheless, half of the cases were non-migrants infected during temporary visits in endemic areas. The patients who were Swedish military personnel were all infected in Afghanistan while stationed there in 2010 [14]. During the same period, other countries with military personnel in Afghanistan reported similar findings [20, 21].

Among the different treatments, cryotherapy had the highest cure rate in the patients with CL. Nonetheless, cryotherapy is not recommended for use on larger lesions (more than 5 cm) and/or lesions on the face or hands. This restriction might have influenced the positive outcome of cryotherapy. The use of cryotherapy is mentioned less frequently in European guidelines for the treatment of CL but seems to have had a positive treatment outcome in our study group. However, more studies are needed to evaluate the efficacy of cryotherapy as a treatment or co-treatment for CL.

Local treatment with intralesional SS had a cure rate of 80% in our study. Although Layegh et al. [22] reported that a combination treatment with intralesional SS and cryotherapy had a better outcome than a single treatment, this combination has only been used in three cases in this study, all with a positive result.

The dose and length of treatment with intravenous SS have varied over time in Sweden. Previously, the WHO recommendations were followed, but as a result of new data [23], treatment is now based on weight and given for a shorter time. There have been some problems in obtaining SS in Sweden in some cases. These problems might have delayed the time to start the treatment in some cases; in other cases, LA was chosen instead.

Different treatment recommendations for LA were used during the study period. Solomon et al. recommended giving LA for five consecutive days at a dose of 3 mg/kg, followed by a sixth dose on day ten [24]. In our study, 22 patients with CL followed this regimen. Twelve patients were cured, and ten patients relapsed, of whom eight had *L. tropica*.

In our study, 15 patients were treated with fluconazole as a first-line treatment; seven (47%) of these patients were cured. Previous studies with fluconazole have shown inconclusive effects on CL caused by *L. major*; with a positive effect reported in studies from high-endemic areas [25, 26], while the same effect was not observed among returning travellers [27]. Five of our seven patients who were cured with fluconazole had an infection caused by *L. major*. Since *L. major* often causes self-healing lesions, it is difficult to evaluate the effect of fluconazole in our study.

The patients with rheumatic diseases and immunosuppressive treatment showed a delay in time to symptoms compared with immunocompetent patients, as described in previous case reports [28, 29]. The patient in this study who was infected with *L. tropica* and treated with TNF inhibitors showed some improvement during treatment with LA but relapsed after treatment. This relapse may have been due to an early restart of immunosuppressive treatment with TNF inhibitors or due to *L. tropica*, which is known to frequently cause relapse. Since there are only approximately forty cases of leishmaniasis occurring in combination with immunosuppressive treatment reported in the litterature [28, 29], and these cases have been caused by various subtypes, there are no general recommendations concerning when to restart treatment with TNF inhibitors. The other two patients with TNF inhibitors in this study were cured after first-line treatment, and they had a different leishmaniasis subtype (*L. donovani*); furthermore, their TNF inhibitor treatments were not restarted. An increasing number of individuals are given immunosuppressive treatments, and when travelling to endemic areas, the number of leishmaniasis cases might increase; therefore, awareness and early identification of leishmaniasis within this group of patients is important.

### Strengths and limitations

This study is a retrospective study based on medical records, and the information was not standardized regarding descriptions of symptoms, definition of cure, treatments, and side effects. Laboratory analyses were not standardized. The number of cases was small, and the subspecies, clinical manifestations and treatments also varied. The different treatments given and the small groups make it difficult to draw any conclusion on the treatment outcome. Additionally, confounding by indication must be taken into consideration when choosing a treatment.

The Swedish healthcare system allows access to medical records, so we were able to include and follow up most of the cases. The Swedish asylum programme offers free care on arrival to Sweden, meaning that all migrant cases have likely been included, even though migrants with no Swedish identification card were lost to follow-up in some cases. The systematic review process was mostly performed by a single investigator (HG 88%, SSK 12%). All microbiological diagnoses were made at the same laboratory (National Reference Laboratory of the Public Health Agency of Sweden).

## Conclusion

The number of leishmaniasis cases diagnosed in Sweden increased between 1996 and 2016, mainly in migrants from endemic countries. Although leishmaniasis is rare in Sweden, patients appear to be diagnosed early and treated according to current European guidelines, resulting in an overall high cure rate.

Although, larger randomized clinical trials are desirable for the evaluation and optimisation of various anti-*Leishmania* drugs, they are difficult to perform in non-endemic areas due to the limited number of cases. Hence, further observational studies on clinical manifestations and treatment outcomes can help us better understand leishmaniasis and optimize disease management in non-endemic countries.

## Additional file

Additional file 1: Country of origin of infection. (PDF 73 kb)

## Abbreviations

CL: Cutaneous leishmaniasis
LA: Liposomal amphotericin
MCL: Mucocutaneous leishmaniasis
PKDL: Post-kala-azar dermal leishmaniasis
SS: Sodium stibogluconate
VL: Visceral leishmaniasis
