# Supplementary figures and images for: Cutaneous, mucocutaneous and visceral leishmaniasis in Sweden from 1996–2016: a retrospective study of clinical characteristics, treatments and outcomes
Source: BMC Infect Dis. 2018 Dec 7;18:632. doi: 10.1186/s12879-018-3539-1 (PMC6286557; doi:10.1186/s12879-018-3539-1)

# Country of origin of infection

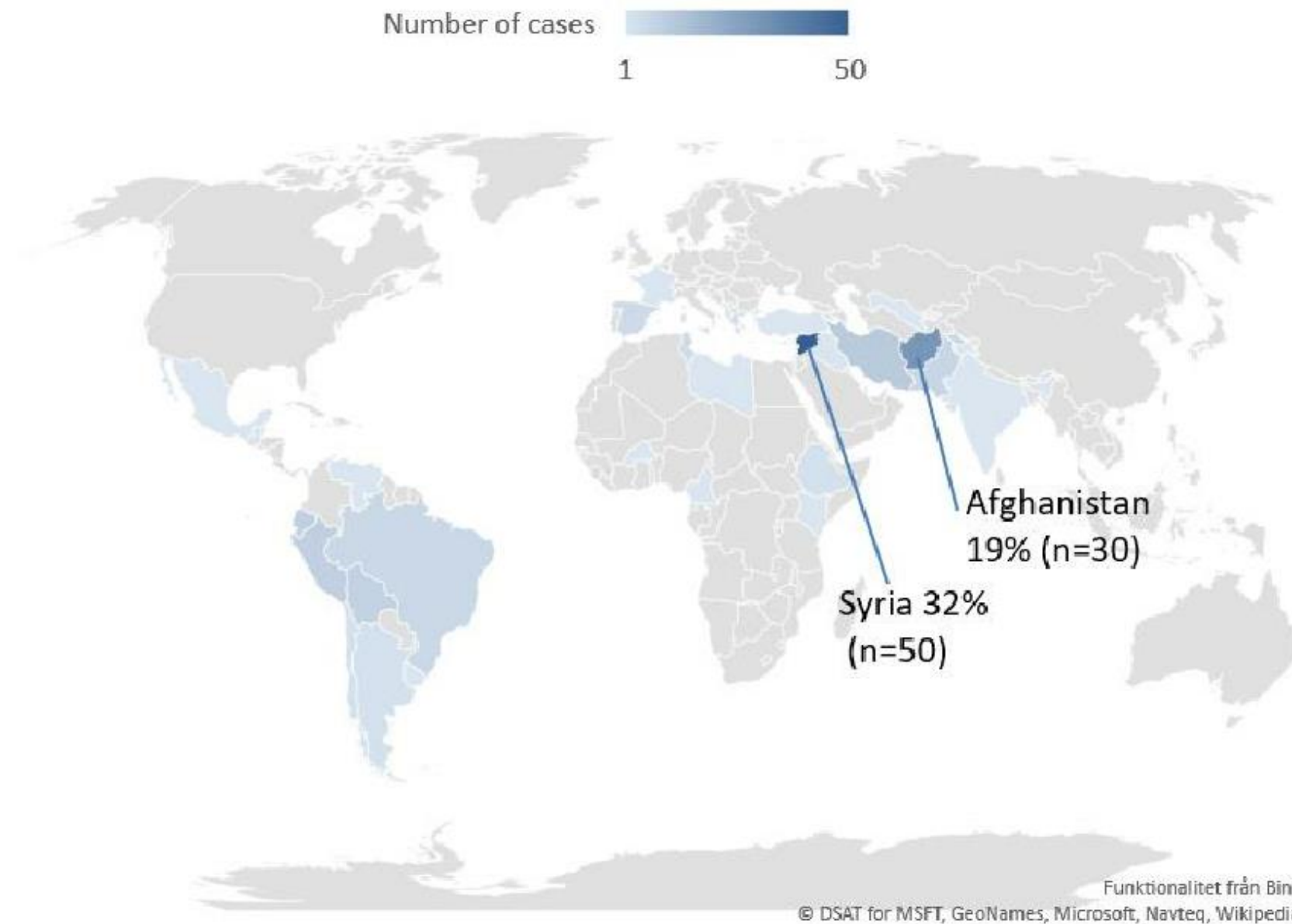

Supplement: Supplementary file 1 — Country of origin of infection. (PDF 73 kb) [file 12879_2018_3539_MOESM1_ESM.pdf]
